# Probabilistic record linkage

**DOI:** 10.1093/ije/dyv322

**Published:** 2015-12-20

**Authors:** Adrian Sayers, Yoav Ben-Shlomo, Ashley W Blom, Fiona Steele

**Affiliations:** 1 School of Clinical Sciences, University of Bristol, Bristol, UK,; 2 School of Social and Community Medicine, University of Bristol, Bristol, UK and; 3 Department of Statistics, London School of Economics and Political Science, London, UK

**Keywords:** Record linkage, epidemiological methods, medical record linkage, bias, data linkage

## Abstract

Studies involving the use of probabilistic record linkage are becoming increasingly common. However, the methods underpinning probabilistic record linkage are not widely taught or understood, and therefore these studies can appear to be a ‘black box’ research tool. In this article, we aim to describe the process of probabilistic record linkage through a simple exemplar. We first introduce the concept of deterministic linkage and contrast this with probabilistic linkage. We illustrate each step of the process using a simple exemplar and describe the data structure required to perform a probabilistic linkage. We describe the process of calculating and interpreting matched weights and how to convert matched weights into posterior probabilities of a match using Bayes theorem. We conclude this article with a brief discussion of some of the computational demands of record linkage, how you might assess the quality of your linkage algorithm, and how epidemiologists can maximize the value of their record-linked research using robust record linkage methods.

Key Messages

- Understanding probabilistic record linkage is essential for conducting robust record linkage studies in routinely collected data and assessing any potential biases.
- Match weights are based on likelihood ratios and are derived from concepts familiar to epidemiologists, such as sensitivity and specificity, and match weights can be converted into probabilities using Bayes theorem.
- Only a basic understanding of conditional probability is required to understand the fundamentals of probabilistic record linkage.

## Introduction

With the increasing use and availability of routinely collected ‘big’ data, it is becoming more useful to undertake research that involves linking data from multiple sources. Therefore, the importance of fully understanding and developing robust record linkage procedures is becoming increasingly necessary, as is fully recognizing and reporting the limitations and biases of the methods used, emphasized by the imminent publication of the record linkage study extension (RECORD^1^^,^^2^) to the STROBE^3^ statement. However, the processes used to link data together are not widely taught, and introductory articles are often complex and relegate the methods to an appendix.^4^ In this article we describe the very common practice of deterministic record linkage and the less common practice of probabilistic record linkage,^5^ using a simple exemplar.

Record linkage can be conceptualized as the process of bringing information from two distinct sources together. However, it also has a number of other uses including building longitudinal profiles, de-duplication of individual records within a single database of records and case re-identification in capture-recapture studies. For simplicity and clarity, we will discuss record linkage in the context of linking data between two databases, although similar methods can be used to link more than two databases.

In general there are two broad types of record linkage methods: (i) deterministic and (ii) probabilistic. Deterministic record linkage is the process of linking information by a uniquely shared key(s). Records are matched if linkage fields agree or unmatched if they disagree. For example, in a longitudinal cohort study, deterministic linkage is often used to link multiple waves of data collection together. Probabilistic record linkage attempts to link two pieces of information together using multiple, possibly non-unique, keys. For example, in a registry-based study, disease events may be linked to mortality data using non-unique first and last name combinations. Despite the apparent simplicity of the task, the process is always complicated by errors in the linkage key(s) or lack of unique key(s) linking both pieces of information together.

In this article we describe: (i) the process of performing record linkage; (ii) pre-merge data cleaning; (iii) the Fellegi-Sunter^5^ statistical framework which underpins much of the research in record linkage; (iv) blocking and stratification; and (v) evaluating linkage errors.

### 

#### Record linkage

For the sake of clarity, we assume a simple scenario where a researcher is attempting to link data from two files. The first file is known as the ‘master file’ (MF) and the second file contains information with which the researcher would like to supplement the master file. This file is known as the ‘file of interest’ (FOI). The information which is used to link the two files together is contained within fields or variables and known as the ‘key’. For pedagogic reasons, we include, as supplementary material (available as Supplementary data at *IJE* online), annotated Stata code which recreates all the analyses described.

#### Deterministic record linkage

Deterministic record linkage is commonly performed in many research studies and assumes there is a known key which links two files together—the MF and FOI, as defined above. The results from a deterministic record linkage procedure will result in two mutually exclusive categories of ‘matched’ and ‘unmatched’ records. Unmatched records can then be further defined as ‘in the master file’ or ‘in file of interest’.

**Figure 1:**
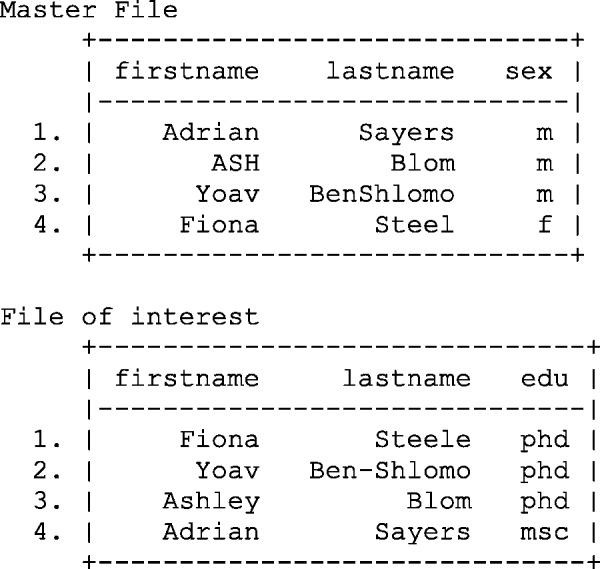
Illustration of two distinct files containing data on sex and education qualification. M, male; F, female; edu, education.

Suppose, for example, that we are interested in investigating the association between an individual’s gender and highest educational qualification, which requires a single data set containing both pieces of information. If we assume gender and education are stored in two distinct files, i.e. ‘the master file’ and the ‘file of interest’, and the linking key is composed of the individual’s first and last name, we can attempt to deterministically link the files. Figure 1 illustrates this scenario: we have four records in each file and we know that there is true one-to-one matching, i.e one record in the master file belongs to one record in the file of interest.

**Figure 2:**
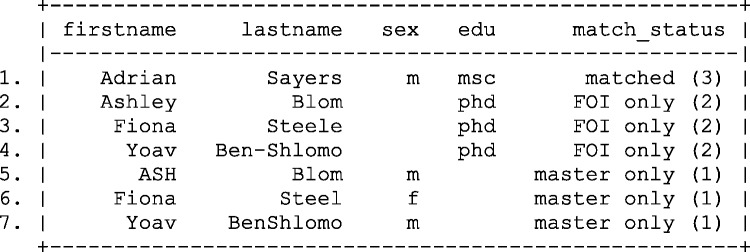
Results of merging two files using first and last name.

Despite the relatively trivial task of linking two data sets by first and last name, the results are somewhat disappointing and there is only one matched record, see Figure 2. Unfortunately, due to selective capitalization, special characters (hyphens, underscores), nicknames, alternative spellings (especially common in ethnic minority names) and spelling mistakes, the record linkage process has been fairly unsuccessful.

However, it is also clear that there is partial agreement between the linking keys of first name and last name. For example, Fiona Steele and Fiona Steel only disagree on a single character in the field last name. Whether the linking fields partially agree or completely disagree is not reflected when conducting a deterministic record linkage. The researcher is then left with the following choices: (i) accept that only a single record is matched between the master file and file of interest; (ii) conduct data cleaning to reduce the heterogeneity in the linkage and reattempt the linkage; or (iii) adopt some form of probabilistic matching.

#### Probabilistic record linkage

Despite the name, the first stage of probabilistic record linkage is not a statistical issue. If you are attempting to link the two files illustrated in Figure 1, you are required to create a file which compares all records in the master file with those in the file of interest.

**Figure 3:**
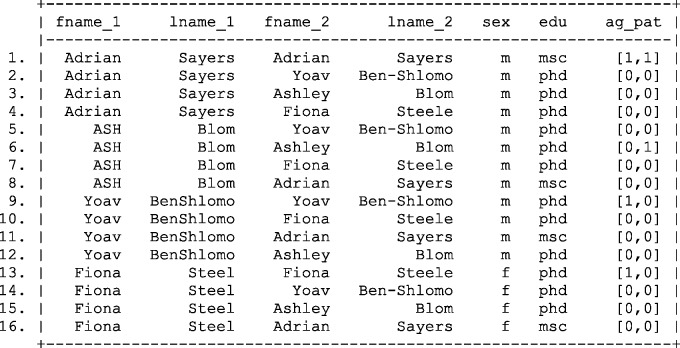
Results of joining two files and calculating simple agreement patterns. Fname, first name; Lname, last name; Ag pat, agreement pattern.

In order to do this, you must first ensure all matching fields are uniquely identifiable across both files. Following the merge (also known as a join) the agreement pattern between the two sets of keys is determined (see Figure 3). The first digit of the agreement pattern (ag_pat) in Figure 3 indicates whether the first name field agrees (coded 1) or disagrees (coded 0), when comparing data from the master file and the file of interest. The second digit relates to agreement on the last name field.

The results of joining the two files and calculating agreement patterns between the linking keys indicates that there maybe more commonality between the two files than previously indicated by deterministic linkage. Whereas only one record is indicated as matching on both the first and the last name fields, there is partial agreement between the first and last name on other records in the data set.

In comparison with deterministic record linkage, the researcher is now in a comparatively informed position. If the choice is to accept only records with identical fields on both the first name and the last name, this will result in a matched data set equivalent to that identified using deterministic linkage. However, the researcher now has the choice of accepting a lower threshold for determining the linkage status of any two records, such as allowing a link to be established on either the first or the last name field.

The simple dichotomy presented by the agreement pattern presented in Figure 3 does not fully reflect the similarity between cases on the first and last name fields. For example, ‘Steele’ and ‘Steel’ only disagree by one character, and therefore to conclude that this field disagrees completely maybe akin to ‘throwing the baby out with the bath water’. The ability to calculate how much any two fields disagree, partially agree or completely agree may be of use.

Assuming that 0 indicates complete disagreement, 1 indicates complete agreement and a value between 0 and 1 indicates partial agreement, a more complex agreement pattern can be constructed. For example, the edit distance^6^^,^^7^ between two variables is one possible method for calculating partial agreement. The edit distance simply counts how many operations (character insertion, deletions and substitutions) are required to turn one string into another. Expressing the edit distance as a proportion of the longest string is one method of calculating the level of disagreement between two variables or, more intuitively, as 1 minus that proportion to indicate how much they agree. For instance, the maximum string length of ‘Steele’ and ‘Steel’ is 6 and a single edit is required to make both fields match; and therefore the edit distance between ‘Steele’ and ‘Steel’ is 1−(1/6) = 0.83. Note that the maximum string length is used to ensure the dis/agreement between fields is constrained within the interval 0 and 1.

**Figure 4:**
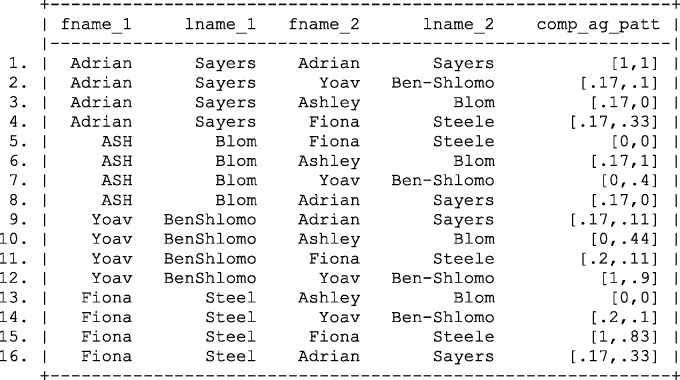
Results of joining two files and calculating complex agreement patterns using the edit distance between first name fields in the master file and the file of interest*.* Fname, first name; Lname, last name; Comp ag pat, complex agreement pattern.

Figure 4 illustrates the results following calculation of complex agreement patterns between first name and last name fields. Calculating a simple edit distance illustrates that small typographical errors such as ‘Steele’ and ‘Steel’ can be compensated for and, if we were willing to accept a threshold of greater than or equal to 0.5 to indicate agreement, we would successfully create two more links. Nevertheless, it is important to note the inequity in the method applied.,For example, ‘Steele’ / ‘Steel’ and ‘Ben-Shlomo’ / ‘BenShlomo’ both differ by a single edit, yet the last name field of ‘Ben-Shlomo’ / ‘BenShlomo’ appears to agree more strongly. Similarly, the entries ‘ASH’ and ‘Ashley’, appear to agree only modestly. If we ignore the capitalization, or unify the case between the fields, a complex agreement pattern of 0.5 would have been calculated.

There are many other methods of comparing the dis-similarity between strings. Common methods include name phonetic algorithms such as SOUNDEX^8^ and NYSIIS^9^^,^^10^. These algorithms attempt to encode names using alpha-numeric or phonetic codes, respectively. However, these methods were originally designed to work with anglicized names, and therefore their suitability in other settings is less clear. There are other more general string encoding methodologies, notably the q-gram approach which divides strings into chunks of size q.^11^ The number of matching q-grams, expressed as a proportion of the number of q-grams in the longest string, can be used to describe the similarity between two string fields. There are also other distance metrics such as the Jaro^12^ or Jaro–Winkler^13^ methods which compare the number of common characters and character transpositions between two strings, with Winkler later amending the method to up-weight similarity at the beginning of the string. Many of the phonetic coding algorithms have been implemented in standard statistical software, e.g. Stata^11^^,^^14^ and R.^11^

Despite the wide variety of methods of comparing strings, any heterogeneity introduced by punctuation, capitalization, abbreviations and alternative spellings emphasizes the need for data cleaning.

#### Pre-merge data cleaning

As emphasized previously, matching on names can be problematic due to typographical differences. Fortunately this problem can be mitigated by data cleaning routines. Examples of common data cleaning procedures can include: (i) changing the case on all the strings; (ii) removing punctuation; (iii) deleting consecutive spaces; (iv) trimming trailing or leading spaces; v5) removing prefixes (Mr/Mrs/Dr/Prof.) and suffixes (II, Jnr, Senior, Esq.); (vi) ignoring middle initials; (vii) looking for transpositions in words (A Sayers, Sayers A); (viii) identifying nicknames (Ash, Ashley) (ix) unifying date formats (31^st^ January 1960, 31/1/1960, 31‐1‐60); (x) checking for transposition in dates (31/1/1960, 1/31/1960); (xi) finding automatically filled dates or dates too far in the future or in the past (1/1/1900, 1/1/2080, 1/1/1880); and (xii) using checksums(i.e. a method which validates an ID using a mathematical algorithm) to find invalid unique identifiers such as those embedded in NHS numbers. Depending on the topic of interest, there may be many other data cleaning procedures which are applicable.

#### Statistical framework underlying probabilistic record linkage

The majority of the statistical framework underlying modern probabilistic record linkage was developed in the late 1950s^1^,^5^ and 1960.^5^ The key features of this framework assume that the master file and the file of interest represent two populations, and that there are some elements which are common to both files.

**Figure 5:**
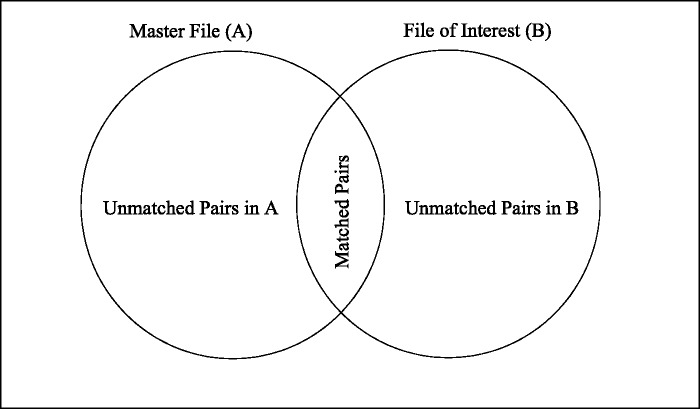
Partitioning of two files into matched and unmatched records.

When a set of all possible matches is created, as in Figure 3 or Figure 4, they theoretically can be partitioned into true matches, indicated by ${\text{M}}_{j}$ (coded 1 for a matched and 0 for an unmatched record), where *j* indexes the potential comparisons from 1 to *J*, and true unmatched records are indicated by $U_{j}=\left(1-{\text{M}}_{j}\right)\;$ (see Figure 5). In practice ${\text{M}}_{j}$ and $U_{j}$ are rarely known.

It is then necessary to assign a numerical value which reflects the (dis-)similarity of the two records. The (dis-)similarity of two records is expressed as the ratio of two conditional probabilities that the two records have the same agreement pattern across the variable of interest.

Suppose we attempt to match two files on first and last name, and we denote the agreement pattern for the *j^th^* comparison by $\gamma_{j}$. The binary agreement indicator for the *i^th^* linkage field of the *j^th^* comparison is denoted by $\gamma_{ij}$, coded 1 for agreement and 0 for disagreement. For example, the agreement indicator for the firstname (i=1) and last name (i=2) fields yields an agreement profile${\text{ γ}}_{j}=\left[\gamma_{1j},\gamma_{2j}\right].\;$ Assuming the true match status of all records is known, the conditional probability that a pair of records has an agreement pattern $\gamma_{j}$, given that it is a true match, is denoted by, $m_{j}=P\left(\gamma_{j}=1|{\text{M}}_{j}=1\right)\equiv P\left(\gamma_{j}|{\text{M}}_{j}\right)$. Similarly, the conditional probability that a pair of records has an agreement pattern $\gamma_{j}$, given they are true unmatched records, is denoted by $u_{j}=P\left(\gamma_{j}=1|{\text{U}}_{j}=1\right)\equiv P\left(\gamma_{j}|{\text{U}}_{j}\right)$. The ratio of $m_{j}$ and $u_{j}$ ($m_{j}/u_{j}$) is a likelihood ratio and forms the basis of the match weight.

The probabilities $m_{j}$ and $u_{j}$ are sometimes referred to as *m-* and *u-probabilities*^5^^,^^16^ and, assuming the agreement between linkage fields are conditionally independent, can be rewritten as $m_{j}=P\left(\gamma_{1j}|{\text{M}}_{j}\right)P\left(\gamma_{2j}|{\text{M}}_{j}\right)$ and $u_{j}=P\left(\gamma_{1j}|{\text{U}}_{j}\right)P\left(\gamma_{2j}|{\text{U}}_{j}\right)$. Conditional independence between the linkage keys of interest appears to be a key assumption in the Fellegi and Sunter formulation.^5^ However, in practice this assumption is likely to be violated. For example, if postal code, street name and county were linkage keys of interest, it is easy to see that if records match on postal code they are more likely to match on street name and county. Despite this limitation, linkage weights are stated to be ‘quite accurate’.^16^

Interpreting *m-* and *u-probabilities* can be difficult. The *m-probability* can be conceptualized as an indicator of data quality. Suppose, for example, that the data error rate (e.g. typographical errors) in records which were truly matches was known, the linkage field was binary (e.g. sex) and this data error rate was approximately 20% in the master file and file of interest. In that case, you would expect 64% (0.8 × 0.8 = 0.64) of matching fields to correctly agree and 4% of matching fields to incorrectly agree (0.2 × 0.2 = 0.04), leading to an *m-probability* of 68% for the sex field. If matching fields are not binary, then the probability of two matching fields incorrectly agreeing is probably closer to zero than 4%. Disagreement in the remaining 32% of pairs of records, i.e. the false negatives, may be due to typographical/data entry errors, missing data and or changes in sex.

The *u-probability* is defined as chance agreement between two records which are truly unmatched. This can be conceptualized and simplified as chance agreement using the following logic. Assume two files (File_Master_, File_FOI_) contain 1000 records each. Then a full comparison between File_Master_ and File_FOI_ will result in 1 000 000 potential comparisons, of which 1000 comparisons can be true matches. Therefore, the 999 000 comparisons are non-matches. As unmatched pairs make up the majority of comparisons, it is often assumed that all comparisons form part of the unmatched set. Assuming that the linkage keys are not unique identifying numbers and have some repetition, it then becomes quite natural to investigate the frequencies within each matching key of File_Master_ and File_FOI_ and how likely it is that a pair of records will match by chance alone.

Both the *m-* and *u-probabilities* can be adjusted depending on the uniqueness (frequencies) of the linking fields. Consider a simple scenario of linkage between two files (File_Master_, File_FOI_) of equal size (N_Master_ = 10,N_FOI_ = 10) without duplicates. Of the 100 comparisons created by joining File_Master_ and File_FOI,_ there will be at most 10 true matches. If the linkage key of interest is surname and there are 7 Smith and 3 Sayers, the *m-probability* of Smith and Sayers is 7/10 = 0.7 and 3/10 = 0.3, respectively. The remaining 90 comparisons are therefore non-matches. We know that there will be 49 comparisons where Smith agrees between the two files of interest; 7 of those comparisons are true links, whereas the remaining 42 are incorrect links. Similarly, there are 9 matches for Sayers of which 3 are correct. The *u-probability* of Smith and Sayers is 42/100 = 0.42 and 6/100 = 0.06, respectively. See

**Figure 6:**
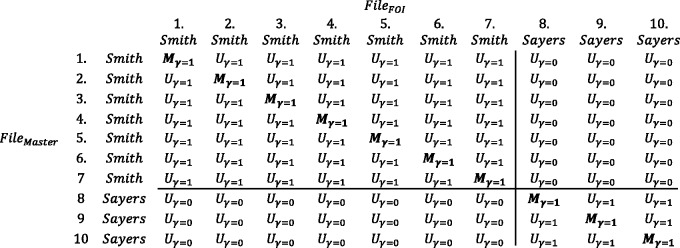
Matrix representation of true match status of two linked files (File_Master_ and File_FOI_) containing varying frequencies of surname $M_{\gamma =1}$ indicates a true matched pair of records where linkage fields agree (bold on the diagonal), ${\text{M}}_{\gamma =0}$ indicates a true matched pair of records where linkage fields disagree, ${\text{U}}_{\gamma =1}$ indicates a true unmatched pair of records where linkage fields agree (the off diagonal elements in the upper left and lower right quadrants), ${\text{U}}_{\gamma =0}$ indicates a true unmatched pair of records where linkage fields disagree (the lower left and upper right quadrants).

Figure 6 for a graphical representation of matched and unmatched status and agreement indicators.

Correspondingly, the likelihood ratios for agreement on ‘Smith’ and ‘Sayers’ are 1.6 (0.7/0.42) and 5 (0.3/0.06), respectively, which indicates that a match on ‘Smith’ is less discriminating than a match on ‘Sayers’.

Formally, if the frequency of names in File_Master_ is defined as $f_{1},\;f_{2},\;\ldots ,\;f_{K}$ and the frequency of names in File_FOI_ as $g_{1},\;g_{2},\;\ldots ,\;g_{K}$, the number of records in file File_Master_ is $N_{Master}=\overset{K}{\underset{k=1}{\sum }}f_{k}$ and File_FOI_ is $N_{FOI}=\overset{K}{\underset{k=1}{\sum }}g_{k}.$ In files of equal sizes and 1:1 matching, the true frequency of matching pairs can be denoted $h_{1},\;h_{2},\;\ldots ,\;h_{K}$, where the number of records in the true match set M is $N_{M}=\overset{K}{\underset{k=1}{\sum }}h_{k}$. Therefore, the frequency-adjusted *m-* and *u-probabilities* are equal to $m_{j}=h_{k}/\;N_{M}$ and $u_{j}=(f_{k}g_{k}-h_{k})/(\;N_{Master}\;N_{FOI}-N_{M})$, respectively.

In Fellegi and Sunters’ original paper they illustrate how to adjust the *m-* and *u-probabilities* for errors and missingness in the linkage fields, and assume *u-probabilities* are an unconditional probability of chance agreement such that $u_{j}=f_{k}g_{k}/N_{Master}N_{FOI}$ irrespective of match status. Despite the apparent simplicity of the calculation of the match weights, either adjusted or unadjusted for their relative frequencies, knowledge of the true match status is required. The true match status of two records is rarely known, and therefore *m-* and *u-*probabilities are either estimated using previous experience, an assumed ‘gold standard’ data set, or by more complex computerized methods.^17^^,^^18^ For example, Harron *et al.* calculated *m-*and *u-probabilities* by deterministically linking a subset of individuals that were matched on either hospital number or NHS number, i.e. they assumed that if a pair of records linked on either field this is a gold standard or at least a reasonable starting point before further refinement.^19^ They then investigated the discordance in other fields which could be used for record linkage outside the subset. For example, if year of birth disagreed in 5% of the linked subset, the *m-probability* for the year of birth would be 0.95.

**Figure 7:**
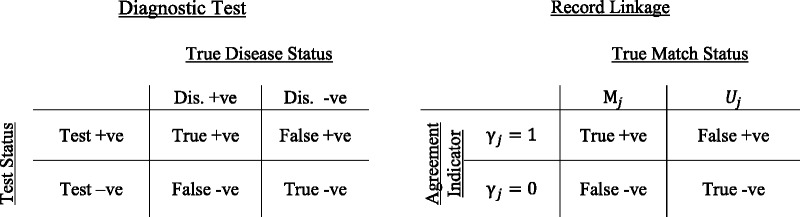
Comparison of results from a diagnostic test against the true disease status and a record linkage against the true match status. Dis, disease.

From an epidemiologist’s perspective, the *m-* and *u-probabilities* are analogous to the results from a diagnostic testing scenario,^20^ see Figure 7. The *m-probability* is equivalent to sensitivity, and the *u-probability* is equivalent to 1 minus the specificity. Furthermore, it is easy to see how the positive predictive value and negative predictive value can be also calculated,^20^ and used to validate the matching process.^21^

After estimating the *m-* and *u-probabilities* of the agreement indicator for the *i^th^* field for the *j^th^* comparison, it is then possible to construct an overall match weight for the *j^th^* comparison, denoted $R\left(\gamma_{j}\right)$. R(γ) is defined using the ratio of the *m-* and *u-probabilities*, where $R\left(\gamma_{j}\right)=\frac{P\left(\gamma_{j}|{\text{M}}_{j}\right)}{P\left(\gamma_{j}|{\text{U}}_{j}\right)}\;$when agreement indicators agree, and $R\left(\gamma_{j}\right)=\frac{1-P\left(\gamma_{j}|{\text{M}}_{j}\right)}{1-P\left(\gamma_{j}|{\text{U}}_{j}\right)}\;$ when agreement indicators disagree. These ratios can be shown to be positive and negative likelihood ratios when agreement indicators agree and disagree, respectively.

Assuming the linkage fields are conditionally independent, the matching weight can be expressed as the ratio of the product of the *m-* and *u-probabilities* across the agreement indicators for the *j^th^* comparison.

(1) $$R\left(\gamma_{j}\right)=\frac{P\left(\gamma_{j}|{\text{M}}_{j}\right)}{P\left(\gamma_{j}|{\text{U}}_{j}\right)}=\frac{\prod_{i}P\left(\gamma_{ij}|{\text{M}}_{j}\right)}{\prod_{i}P\left(\gamma_{ij}|{\text{U}}_{j}\right)}$$

However, it is common to use logarithms (base 2)^15^ of this ratio as this simplifies the calculation^5^ and eases the interpretation of the match weights, so that a 1 unit increase in ${\text{log}}_{2}R\left(\gamma_{j}\right)\;$ represents a doubling in the likelihood ratio for a matched pair of records.

(2) $$\log_{2}R\left(\gamma_{j}\right)=\underset{i}{\sum }\log_{2}\left(\frac{P\left(\gamma_{ij}|{\text{M}}_{j}\right)}{P\left(\gamma_{ij}|{\text{U}}_{j}\right)}\right)$$

**Figure 8:**
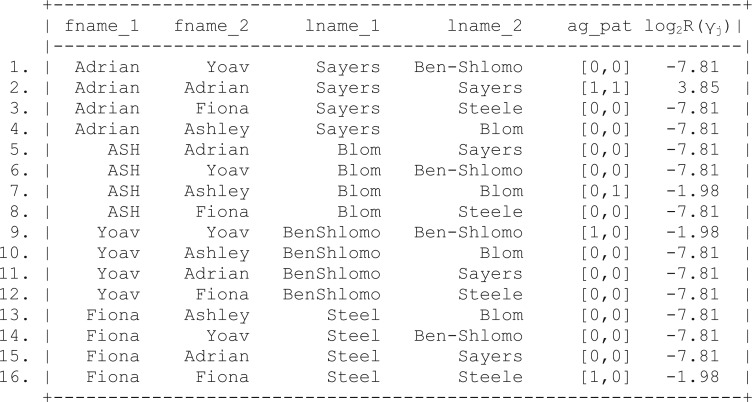
Calculation of simple agreement weights, ${\text{ log}}_{2}\text{R}\left(\gamma_{\text{j}}\right)$, using the Fellegi and Sunter record linkage framework.^5^ Fname, first name; Lname, last name; Ag pat, agreement pattern.

Applying this framework to the simple agreement patterns displayed in Figure 3, using *m*- and *u-probabilities* of 0.95 and 0.25 for first and last name fields, respectively, yields the weights shown in Figure **8**.

Furthermore, the match weights can be adjusted for complex agreement patterns, $R\left(\gamma_{j}^{\prime }\right)$. Previously the agreement indicators $\left(\gamma_{ij}^{}\right)$ are simply coded as either 0 (disagreement) or 1 (agreement), whereas complex agreement indicators $\left(\gamma_{ij}^{\prime }\right)$ can take on any value between 0 (complete disagreement) and 1 (complete agreement) where values greater than 0 and less than 1 indicate partial agreement. The match weights based on complex agreement patterns are calculated by subtracting the difference between the match weights when the agreement indicators agree and disagree, multiplied by 1 minus the complex agreement pattern ($\gamma_{ij}^{\prime }$).

(3) $$\log_{2}R\left(\gamma_{j}^{\prime }\right)=\underset{i}{\sum }{\text{log}}_{2}\left[\left(\frac{P\left(\gamma_{ij}|{\text{M}}_{j}\right)}{P\left(\gamma_{ij}|{\text{U}}_{j}\right)}\right)-\left(\frac{P\left(\gamma_{ij}|{\text{M}}_{j}\right)}{P\left(\gamma_{ij}|{\text{U}}_{j}\right)}-\frac{1-P\left(\gamma_{ij}|{\text{M}}_{j}\right)}{1-\left(\gamma_{ij}|{\text{U}}_{j}\right)}\right)\left(1-\gamma_{ij}^{\prime }\right)\right]$$

**Figure 9:**
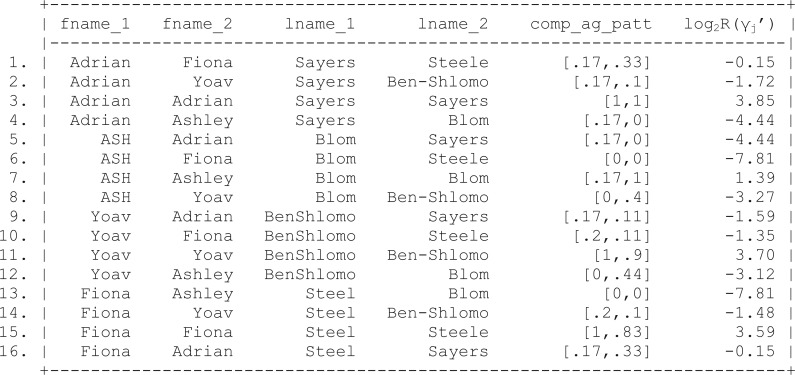
Calculating of complex agreement weights, ${\text{ log}}_{2}\text{R}\left(\gamma_{\text{j}}^{\text{′}}\right)$, using the Fellegi and Sunter record linkage framework. Fname, first name; Lname, last name; Ag pat, agreement pattern.

Applying the modified weight calculation to the complex agreement patterns presented in Figure 4 results in the refined weight calculation shown in Figure 9.

**Figure 10:**
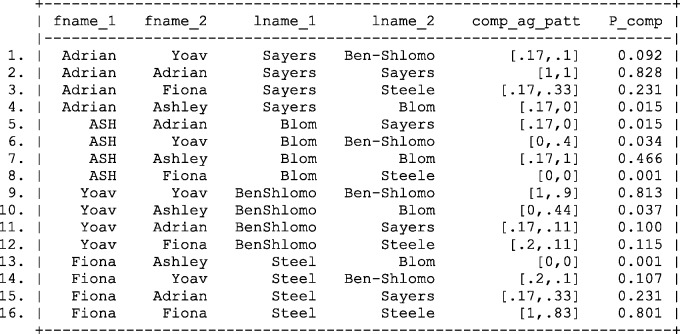
Probabilities of links based complex agreement weights, ${\text{ log}}_{2}\left(\text{R}\left(\gamma_{\text{j}}^{\text{′}}\right)\right)$ calculated using Bayes theorem. Fname, first name; Lname, last name; Comp ag pat, complex agreement pattern.

Despite the somewhat difficult interpretation of the linkage weights, it is very clear which records are likely to be a match. For example, we can see that records 3, 7, 11 and 15 are 4, 5.83, 5.05 and 3.74 times more likely to match than the next nearest matching record, respectively.

The final operation is to define two thresholds which classify the potential links into three categories: links, non-links and potential links. It is possible to generate two different thresholds using the distribution of linkage weights, $log_{2}R\left(\gamma_{j}\right)$ or $log_{2}R\left(\gamma_{j}^{\prime }\right)$, but they often prove difficult to interpret. Therefore a number of authors have pointed out that it may be preferable to define linkage status on the probability scale. Using Bayes theorem, it can be shown how $R\left(\gamma_{j}\right)$ or $R\left(\gamma_{j}^{\prime }\right)$ and the group prior odds of a match, $P{\left(M\right)}_{prior}/(1-\;P{\left(M\right)}_{prior})$, can be used to estimate the posterior odds of a match,^22^ which in turn can be converted into probabilities.

The prior probability of a match is defined as:

(4) $$P{\left(M\right)}_{prior}=\frac{N_{expected.matches}}{N_{Master}}\cdot \frac{1}{N_{FOI}},$$

where $N_{expected.matches}$ is the number of anticipated matches between the master file and the file of interest and $N_{Master\;}$and $N_{FOI}\;$are the total number of records in each file. The posterior odds ratio, $\frac{P\left({\text{M}}_{j}|\gamma_{j}\right)}{P\left({\text{U}}_{j}|\gamma_{j}\right)}$, is defined as the product of the likelihood ratio and the prior odds of a match:

(5) $$\frac{P\left({\text{M}}_{j}|\gamma_{j}\right)}{P\left({\text{U}}_{j}|\gamma_{j}\right)}=\frac{P\left(\gamma_{j}|{\text{M}}_{j}\right)}{P\left(\gamma_{j}|{\text{U}}_{j}\right)}\cdot \frac{P{\left(M\right)}_{prior}}{P{\left(U\right)}_{prior}}$$

Therefore the probability that any two records are a match can be calculated as follows:

(6) $$P{\left(M\right)}_{Posterior}=\frac{\frac{P\left({\text{M}}_{j}|\gamma_{j}\right)}{P\left({\text{U}}_{j}|\gamma_{j}\right)}}{1+\frac{P\left({\text{M}}_{j}|\gamma_{j}\right)}{P\left({\text{U}}_{j}|\gamma_{j}\right)}}$$

Applying these results to the complex agreement weights presented in Figure 9 results in the posterior probabilities of a match shown in Figure 10.

Despite exact agreement of linkage fields, the probability of linking records between the master file and the file of interest is less than 1,reflecting the possibility of inconsistencies in the data quality and chance agreement. However, by eyeballing the data, it is clear that the majority of correct links are identified with probability greater than 0.8.

The exact placements of thresholds used to define link status can be a matter of trial and error.^23^ The need to maximize sensitivity of detecting matches will undoubtedly necessitate more clerical review of links compared with that of a threshold which optimizes specificity. The choices of optimizing sensitivity or specificity will likely depend on the questions being asked and concerns with regard to potential misclassification.

#### Blocking and stratification

Despite the trivial example presented previously, it is very easy to see how the size of linked datasets can quickly expand. Even with a modestly large master file and file of interest of 10 000 individuals, the resulting linked file would result in 100 000 000 potential links. In projects using routinely collected data, the number of individuals of interest can be 1 × 10^6^ or more. Therefore, the use of blocking or stratification is employed. This process involves splitting the database into smaller blocks or strata, which was originally described as the ‘restriction of explicit comparisons to a subspace’.^5^ For example, if the project of interest is national, you may decide to block by region. This simply means that you only look for matching records within a region. Partitioning the data set greatly reduces the comparison space; for example, attempting to perform linkage between two data sets each with 10 000 individuals equally distributed across 10 regions would result in a file with 10 × 10^6^ potential links, in contrast to the unblocked comparison which would result in 10 × 10^7^ potential links. Nevertheless, when blocking there is a clear trade-off between the size of the blocks and the ability to fully explore the data set looking for potential matches, with the explicit assumption that individuals not in the block will not be a match.

#### Reporting linkage errors

Following the creation of a linked data set, it is important to consider the quality of linkages and how this might influence your results^24^^,^^25^ i.e. how many incorrect links you have made, how many correct links you have missed and what bias this may cause. Attempting to do this seems somewhat counter-intuitive, because if you a priori knew the true linkage status of a record, there would have been no need to have conducted a probabilistic linkage.

There are a number of different approaches which can be used to quantify the rate of linkage errors including: (i) comparison with a gold-standard sub-sample; (ii) sensitivity analysis; (iii) comparison of linked and unlinked data; and (iv) identification of implausible matches.

Using a gold-standard sub-sample is probably the most intuitive method of establishing linkage errors. Comparing the probabilistically linked data set to the gold-standard sub-sample will give rise to a simple 2 × 2 table of linkage errors. Following creation of the 2 × 2 table, simple statistics such as sensitivity, specificity and positive/negative predictive values can be calculated and reported.

Structured sensitivity analyses can also be used to see how the changes to the *m-* and *u-*probabilities influence the number of potential links. Comparing linked and unlinked data can also be useful in establishing if some groups of records are easier to link than others. For example, assuming there are some common fields not used as linkage keys, such as socioeconomic status (SES), it is possible to compare the linkage rates within the SES group of the master file. Similarly, investigating how linkage rates vary across time might be a useful indicator of time-dependent biases.

Identifying implausible matches may only be possible in specific scenarios. Suppose, for example, that probabilistic linkage was being used to ascertain patient mortality within routinely collected medical records, and a trajectory indicated the following mortality pattern: alive, dead, alive. There may be some question about the quality of the linkages or the veracity of the source data.

#### Conclusion

We have described the process underlying deterministic and probabilistic record linkage using a simple exemplar. Despite the apparent complexity of probabilistic linkage, it can be broken down into a relatively small number of simple data manipulation operations with relatively little statistical knowledge. Furthermore, the statistical principles underpinning the weight calculations used to define links in probabilistic linkage can be derived from Bayes’ theorem, which may be covered on epidemiology courses and only requires a rudimentary understanding of conditional probability.

Using a simple exemplar we have illustrated the critical steps and assumptions that underpin probabilistic record linkage. These include: (i) the inequity of the edit distance when comparing long and short strings; (ii) the assumption of conditional independence when calculating the match weights; (iii) the choice of block size which influences the computational burden of the linkage exercise; (iv) the choice of selection thresholds before accepting or rejecting pairs of records as links or non-links, and those requiring clerical review; and (v) the somewhat arbitrary pre-merge data cleaning processes that occur in the hope of finding more matching records.

The benefits of probabilistic record linkage are simple (reduced missing data, improved classification using the linked variables of interest). However, despite the simplicity of the exemplar, there are many complex issues of current research in the record linkage field including privacy preserving record linkage,^22^ efficient analysis of record linked data sets^26^ and efficient automated selection of matched and non-matched records using an EM algorithm.^27^

Record linkage, whether probabilistic or deterministic, will become increasingly important as the breadth and scope of routinely collected data rapidly expand. We have illustrated that it is simple to conduct robust probabilistic record linkage using standard statistical software, and that sensitivity of results can be easily explored using different matching assumptions. Furthermore, probabilistic record linkage has the potential to maximize the value of routinely collected data by improving the linkage between the linked files of interest, which in turn will reduce the volume of missing data and improve the classification within the linked variables of interest, thereby strengthening the inferences from linkage studies.

## Supplementary Data

Supplementary data are available at *IJE* online.

## Funding

A.S. is funded by an MRC Fellowship (MR/L01226X/1).

## Supplementary Material

Supplementary Data

## Appendix

Assuming we can estimate the probability of ${\text{M}}_{j}$ and $U_{j}$ we can use Bayes’ theorem to derive the odds ratio of a match.

Using Bayes’ theorem the probability of match and unmatched records conditional on the agreement pattern is defined as follows.

(A1) P(Mj|γj)=P(γj|Mj)P(Mj)P(γj)P(Uj|γj)=P(γj|Uj)P(Uj)P(γj)

The ratio of $P\left({\text{M}}_{j}|\gamma_{j}\right)$ and $P\left({\text{U}}_{j}|\gamma_{j}\right)$ is referred to as the posterior odds, and can be written as

(A2) $$\frac{P\left({\text{M}}_{j}|\gamma_{j}\right)}{P\left({\text{U}}_{j}|\gamma_{j}\right)}=\frac{\frac{P\left(\gamma_{j}|{\text{M}}_{j}\right)P\left({\text{M}}_{j}\right)}{P\left(\gamma_{j}\right)}}{\frac{P\left(\gamma_{j}|{\text{U}}_{j}\right)P\left({\text{U}}_{j}\right)}{P\left(\gamma_{j}\right)}}$$

Following rearrangement

(A3) $$\frac{P\left({\text{M}}_{j}|\gamma_{j}\right)}{P\left({\text{U}}_{j}|\gamma_{j}\right)}=\frac{P\left(\gamma_{j}|{\text{M}}_{j}\right)P\left({\text{M}}_{j}\right)P\left(\gamma_{j}\right)}{P\left(\gamma_{j}|{\text{U}}_{j}\right)P\left({\text{U}}_{j}\right)P\left(\gamma_{j}\right)}$$

and simplification of like terms (cancelling $P\left(\gamma_{j}\right)$) results in the following expression

(A4) $$\frac{P\left({\text{M}}_{j}|\gamma_{j}\right)}{P\left({\text{U}}_{j}|\gamma_{j}\right)}=\frac{P\left(\gamma_{j}|{\text{M}}_{j}\right)P\left({\text{M}}_{j}\right)}{P\left(\gamma_{j}|{\text{U}}_{j}\right)P\left({\text{U}}_{j}\right)}$$

The ratio $\frac{P\left(\gamma_{j}|{\text{M}}_{j}\right)}{P\left(\gamma_{j}|{\text{U}}_{j}\right)}\;$ can be shown to be a likelihood ratio, and the ratio $\frac{P\left({\text{M}}_{j}\right)}{P\left({\text{U}}_{j}\right)}$ is known as the prior odds.
